# Automated Generation of Clinical Reports Using Sensing Technologies with Deep Learning Techniques

**DOI:** 10.3390/s24092751

**Published:** 2024-04-25

**Authors:** Celia Cabello-Collado, Javier Rodriguez-Juan, David Ortiz-Perez, Jose Garcia-Rodriguez, David Tomás, Maria Flores Vizcaya-Moreno

**Affiliations:** 1 Department of Computer Technology, University of Alicante, 03080 Alicante, Spain; ccabello@dtic.ua.es (C.C.-C.); jrodriguez@dtic.ua.es (J.R.-J.); dortiz@dtic.ua.es (D.O.-P.); 2 Department of Computer Languages, University of Alicante, 03080 Alicante, Spain; dtomas@dlsi.ua.es; 3 Unit of Clinical Nursing Research, Faculty of Health Sciences, University of Alicante, 03080 Alicante, Spain; flores.vizcaya@ua.es

**Keywords:** text summarization, healthcare, multimodal data, audio sensors, transformers

## Abstract

This study presents a pioneering approach that leverages advanced sensing technologies and data processing techniques to enhance the process of clinical documentation generation during medical consultations. By employing sophisticated sensors to capture and interpret various cues such as speech patterns, intonations, or pauses, the system aims to accurately perceive and understand patient–doctor interactions in real time. This sensing capability allows for the automation of transcription and summarization tasks, facilitating the creation of concise and informative clinical documents. Through the integration of automatic speech recognition sensors, spoken dialogue is seamlessly converted into text, enabling efficient data capture. Additionally, deep models such as Transformer models are utilized to extract and analyze crucial information from the dialogue, ensuring that the generated summaries encapsulate the essence of the consultations accurately. Despite encountering challenges during development, experimentation with these sensing technologies has yielded promising results. The system achieved a maximum ROUGE-1 metric score of 0.57, demonstrating its effectiveness in summarizing complex medical discussions. This sensor-based approach aims to alleviate the administrative burden on healthcare professionals by automating documentation tasks and safeguarding important patient information. Ultimately, by enhancing the efficiency and reliability of clinical documentation, this innovative method contributes to improving overall healthcare outcomes.

## 1. Introduction

The digitization and automation of healthcare systems has been an urgent need for decades, which has increased in the wake of the COVID-19 pandemic. The introduction of artificial intelligence (AI) in hospitals has been gradual compared to other fields such as industry and finance, where this technology is the order of the day. This urgency is due to the extended occupational stress burnout suffered by doctors within the healthcare scope [1,2]. This condition is a response to chronic work stress that negatively affects cognitive, emotional, and attitudinal factors and can generate negative behaviors towards work and people related to it, accompanied by psychological alterations. A recent study [3] on the prevalence of burnout in primary care (PC) doctors in Madrid concluded that 69.2% are affected by burnout. Among the causes of burnout in Spanish doctors are work overload, work-related stress, lack of institutional support, difficulty in reconciling work and personal life, and pressure to provide care. In addition, the COVID-19 pandemic exacerbated these problems, as doctors have been emotionally stressed due to the healthcare crisis. Burnout in doctors not only affects the health and well-being of the professional but can also have negative consequences on the quality of medical care and patient safety. Therefore, it is important that measures are taken to prevent and treat burnout in the medical profession. In addition to reducing workload and providing institutional resources for stress management and work–life balance, the introduction of new technologies that decrease bureaucratic burden and support healthcare workers in an activity whose systems are currently on the brink of collapse is essential.

Given the current healthcare context, AI, and specifically deep learning methods, can be leveraged to mitigate these challenges. For this reason, this study proposes a framework to automatically transcribe and summarize the most remarkable information from doctor–patient interactions. To implement the pipeline, a comparison of well-known models is utilized as the basis. This comparative analysis aims to identify the most effective techniques for accurately capturing and synthesizing the nuances of medical conversations. The system was evaluated using a dataset specifically created for this study, which contains professional summaries of patient–doctor interactions. This dataset serves not only as a test bed for assessing the system’s performance but also as a valuable resource for further research in automated medical documentation. The evaluation showed the system’s positive performance capabilities, highlighting its potential in accurately extracting and condensing key information from complex clinical dialogues. Despite the limitations posed by the experimentation, such as the potential for bias in the dataset and the inherent challenges in processing natural language, the results are promising.

This system can potentially be applied in healthcare centers to reduce the workload of healthcare professionals, thereby allowing them to devote more time to direct patient care. Furthermore, by improving the efficiency and accuracy of medical documentation, the system can enhance the capacity and quality of assistance that healthcare centers provide to society. This improvement could lead to better patient outcomes, increased satisfaction, and a reduction in documentation errors. Despite the great benefits that this system can offer, it should be remembered that this system is intended to be used as an assistant, not as an automated decision-making system. The supervision of the summaries generated by a doctor is essential to avoid negative consequences and misunderstandings. The main contributions of this work are summarized as follows:

- Development of a NLP and ASR based system to transcript and summarize patient–doctor interactions in a primary attention center;
- Creation of a validation set composed of real conversations and their associated summaries created by healthcare professionals;
- Reduction of the bureaucratic burden of healthcare personnel for improving the quality of their daily work life.

This article is organized into four additional sections. Section 2 provides a review of work related to the fields of NLP and ASR, which are relevant for the development of this study. Section 3 shows the basis components used for the experimentation. Section 4 explains the complete experimentation and results. Finally, Section 5 presents the final conclusions of the study.

## 2. Related Work

In this section, a review will be conducted of the work covering state-of-the-art methods and technologies utilized within the fields of ASR and text summarization. The methods and resources described in the following subsections will form the foundation of this study’s contribution.

### 2.1. Natural Language Processing

Natural language processing (NLP) and automatic speech recognition (ASR) are highly studied fields within the deep learning community, offering various methods that will be employed to achieve the objectives of this study. NLP utilizes AI, linguistics, and data science to enable computers to perform tasks related to understanding human language, both in written and spoken forms [4]. It organizes data through entity recognition and word pattern identification, employing techniques such as tokenization (term extraction), stemming (identifying word roots), and lemmatization (obtaining the dictionary form of a word). The study of NLP can be divided into two main areas: natural language understanding [5], which enables machines to comprehend and analyze natural language by extracting concepts, entities, or keywords, and natural language generation [6], focused on generating textual content.

Within natural language understanding, linguistics plays a pivotal role, making fields such as lexical or semantic analysis crucial. There exists a broad range of tasks in this field, including part-of-speech tagging [7], aimed at categorizing words in a text according to their part of speech based on the word’s definition and context, and sentiment analysis [8], which seeks to determine the overall sentiment of a document, sentence, or aspect thereof, attempting to identify the author’s attitude through the conveyed feelings, judgments, or evaluations.

In the realm of natural language generation, a variety of tasks can be observed, such as language modeling, aimed at predicting the next word or sequence of words in a sentence given the preceding context, and serving as the foundation for many other text generation tasks; translation, which involves converting text from one language to another while preserving the original text’s meaning and context; and summarization [9], which aims to condense a longer text into a shorter, coherent summary while retaining the most crucial information. This aspect will be a core focus of this study, and its subtypes and common implementations will be further explained in subsequent sections. On the other hand, ASR is recognized as a subfield of NLP aimed at enabling computers to transcribe spoken language into text. This field has also been intensively explored for the development of various applications such as virtual assistants [10] and efficient human transcription systems [11].

### 2.2. Text Summarization

The task of text summarization [9] is directed towards generating a shortened text that retains all the relevant information from the original document, with the aim of enhancing text processing speed without losing the context of the original text. Over the last decade, various research trends in automatic text summarization have been explored [12], but the primary research lines are abstractive and extractive methods.

Extractive summarization [13] focuses on selecting key phrases from the input document, which are then concatenated to form a summary of a specific length. One advantage of this type of summary is its objectivity. To identify key phrases, there are some methods that are usually considered. Some of these methods include the term frequency–inverse document frequency (TF-IDF), which highlights prominent terms based on term frequency; the cue-based method, which evaluates words that would affect the weight of the corresponding phrase or word (adjectives accompanying the word “error” (“significant” or “insignificant”) may be crucial in determining the importance of the noun); the analysis of words from the title or heading, which are assumed to be more relevant to the summary; or the discard of short phrases, which often contain little information.

In contrast, abstractive summarization [14] is created from a paraphrase of the text, aiming to produce a concise and coherent summary that captures the essential meaning of the original text without solely relying on extracting fragments from the original text. These methods have evolved from classical approaches, where text templates were used, to the application of sophisticated neural approaches based on deep learning. Abstractive summarization techniques are classified into three different categories, reflecting the key advances introduced within these methods:

- **Template-driven Generation:** This technique utilizes predefined templates [15] or sentence structures to generate summaries. These templates may have blank spaces or markers filled with relevant details extracted from the source material. Although this method is less flexible and innovative, it proves useful in specific fields characterized by well-defined text formats.
- **Language Model-based Generation:** This approach relies on deep learning architectures like Seq2Seq models [16], trained on extensive datasets to understand and encapsulate text structures and semantics.
- **Language Generation with Attention Mechanisms:** Employing models that integrate attention mechanisms [17], such as Transformer models [18], this method focuses on significant segments of the source text during summary creation. Attention mechanisms allow the model to assign importance and relevance to various text segments during summary generation, enhancing coherence and salience in the final summary output.

The automatic generation of text summaries finds application across a wide array of domains, such as media for creating brief summaries of articles and live broadcasts, in research to condense lengthy academic articles, and in search engines to provide summaries of web page contents.

Within healthcare, automatic text generation already has significant applications, like summarizing clinical records [19] or automatically generating medical reports from doctor–patient interactions [20], which is the main focus of this work. Within this specific topic, work such as that presented in [21,22] is outstanding, where ASR models are used alongside sequence-to-sequence models to generate textual summaries from speech input data. It is crucial to note that automatic summarization in the medical field requires a careful and precise approach due to the critical nature of medical information.

### 2.3. Automatic Speech Recognition

ASR is the task aimed at obtaining textual information from audio inputs. Over the past decade, the accuracy of ASR systems has significantly improved due to the adoption of deep neural network-based hybrid modeling [23,24]. This approach, which replaced the traditional Gaussian mixture model [25] for assessing acoustic probability, retains key components such as the acoustic model, language model, and lexicon. A major advancement was the transition from hybrid modeling to end-to-end (E2E) modeling [26], which directly translates an input speech sequence into an output sequence of tokens using a single network.

E2E modeling offers several significant advantages over the traditional hybrid model. First, the entire network is optimized using a single objective function specific to an ASR task, in contrast to traditional hybrid models that optimize individual components separately, potentially missing a global optimum. Second, the E2E model simplifies the ASR process by directly generating letters or words, whereas designing traditional hybrid models is complex and requires deep expertise from specialists with years of experience in the ASR field. Additionally, as ASR utilizes a single network, the E2E model is more compact than the traditional hybrid model, making it easier to implement on devices with limited resources. The trend in ASR is shifting towards state-of-the-art deep learning models, such as Transformer-based models [27], leading to significant improvements in accuracy and applicability across a wide range of applications, including virtual assistants, automatic transcription, real-time translation, and beyond.

Despite the positive results obtained by end-to-end models in the ASR field, the application of these models still faces challenges [28] that are crucial to address to expand the technology’s use in healthcare. A primary challenge is protecting against adversarial attacks [29] targeting speech, where imperceptible perturbations are strategically added to input samples using optimization algorithms to deceive the classifier into making incorrect decisions. These attacks can significantly degrade the performance of advanced deep neural network-based systems. Additional challenges include the scarcity of speech data needed to train large neural networks and interoperability issues in current healthcare systems, where medical data is isolated and fragmented across different hospitals or laboratories due to privacy restrictions.

## 3. Methodology

**Figure 1:**
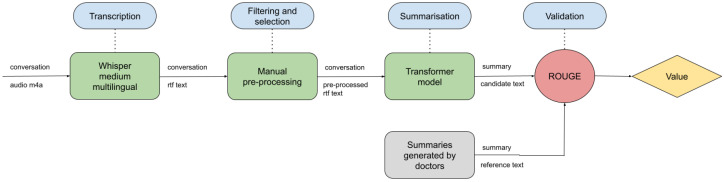
Scheme of the complete proposed system.

Throughout this section, the components and platforms used to construct the proposed method are explained. The method is composed of three main processing steps: First, the input audio will be the input of an ASR model, which will generate a transcription of the audio file. Second, a manual cleaning stage will be carried out to remove noise and adapt the text to the next processing stage. Finally, a summarization model will be used to obtain the summary of the cleaned transcription. The Transformers library [30] has been used as a support platform to make use of state-of-the-art summarization models. Moreover, within this section, the validation set used to test the effectiveness of the model is explained. The complete pipeline is shown in Figure 1.

### 3.1. Hugging Face Transformers

The Transformers library [30] from Hugging Face (https://huggingface.co/, accessed on 22 March 2024) is one of the most powerful tools currently available for the design and use of Transformer-based systems. Within this library, researchers can find an easy API to train and fine-tune models covering a wide variety of tasks, from NLP or computer vision tasks to activities that involve the use of multimodal data. One of the main goals of this library is to bring the Transformers architecture to everyone, allowing researchers and engineers to leverage this powerful architecture without the need to develop and train these models from scratch, which is time-consuming and practically impossible for individuals due to the computational requirements needed to train models as large and complex as the ones available in the library.

The Transformers library stands out for its broad selection of models designed for various languages and domains. Notable examples include BERT [31], GPT [32], and RoBERTa [33], among others. Each of these models has undergone extensive training on large datasets to excel in specific tasks. These models, and others within the Transformers library, are pivotal not only for advancing academic research but also for driving innovation in industries reliant on understanding and generating human language.

### 3.2. Dialogues Dataset

Due to the scarce data available involving doctor–patient interactions, the project required creating a compact collection of M4A format audio recordings featuring doctor–patient dialogues. These recordings were captured during primary healthcare consultations, accompanied by duly signed consent forms from both the patient and the medical practitioner. Moreover, two healthcare professionals contributed to creating summaries for each of the conversations.

Out of these recordings, 14 dialogues were chosen based on criteria such as the length of the dialogues, the clarity of the exchanges, and the sound fidelity. This strict selection is done to ensure the quality of the conversations used as the basis for verifying the system. Moreover, the usage of genuine dialogues adds authenticity and realism crucial for accurately evaluating the system’s performance in real-world scenarios. Alongside each dialogue, two reference conversation summaries are provided. These summaries were collected by the doctor and the nurse in charge of the consultation during their interactions with the patients. Two summaries were collected to ensure the reliability of the information provided while also offering different perspectives on the dialogue. To improve the quality and comprehension of the summaries, they were reviewed and corrected for crucial spelling and grammatical errors at the end of the consultations. These summaries will represent the ground truth to be compared with the summaries created by the proposed system. As the complete dataset was collected in a Spanish primary healthcare consultation, all the data are in the Spanish language.

Regarding the distribution of the data, the selected dialogues reflect the natural distribution of topics covered in primary healthcare consultations, where the majority of the sessions focus on reviewing results from various tests or monitoring the status of any injury or illness. This distribution is as follows. Seven out of the fourteen conversations are about test results, which are consultations aimed at reviewing the results obtained from tests such as X-rays, ultrasound scans, or blood tests. Five out of the fourteen are about diseases status reviews, which are recurring consultations aimed at tracking the progression of a disease or an injury. Finally, the last two conversations are about newly detected diseases, which are consultations in which the patient informs the doctor about a newly detected illness or injury in order to receive assistance on how to address the problem. On the other hand, Table 1 presents statistics about the content of the data in terms of the number of sentences, words, and turns. The turns represent the total number of different doctor–patient interactions in the complete dataset.

### 3.3. ASR Component

In the first stage of the system, the input audio file is provided to an ASR model, which transcribes the audio into text. The model selected for this audio-to-text task is Whisper [27], trained with almost 680,000 hours of multilingual data and supervised multitasking. This extensive dataset ensures high effectiveness in applying diacritical marks, transcribing noise-affected audio, and recognizing specialized language.

The Whisper model employs a comprehensive, end-to-end approach, utilizing Transformer encoder–decoder modules. Each audio piece is reprocessed to generate a Mel spectrogram representation, which is then processed by the encoder through two convolutional layers and the GELU activation function. Subsequently, sinusoidal positional embeddings are added to the result, before the application of the Transformer encoder modules. The decoder uses pre-learned positional embeddings and linked input–output token pair representations.

Prediction initiation starts with a “start of transcript” token, initially determining the text’s language, indicated by unique tokens for each of the 99 languages in the training set (if a multilingual model is used). If an audio segment contains no spoken words, the model can predict a “no speech” token. Next, the model identifies the task—either “transcribe” for transcription or “translate” for translation—and decides on including or excluding timestamps (for time alignment and subtitles) via a “no timestamps” token, before proceeding to output generation. The process concludes with an “end of transcript” (EOT) token.

Concerning the models that are accessible, there exist multiple Whisper models in various sizes, offering excellent scalability. For the transcription of dialogues, the medium multilingual model was chosen, given that the dialogues were in Spanish and monolingual models are only available for English. In Table 2, a summary of the different Whisper models available and their requirements is shown. Preliminary tests were conducted on both the medium and large models, which showed nearly identical transcription outcomes for the same text. Due to the large model’s excessive video random-access memory (VRAM) requirements, which would unnecessarily slow down processing without significantly enhancing transcription quality, the medium model was preferred for this project.

### 3.4. Transcription Processing

Given the complex nature of conversations in healthcare, characterized by varied structures and themes within primary care settings, and the limited dataset size, which complicates categorization efforts, it was decided to perform some basic manual preprocessing of the dialogues. The preprocessing steps applied to the dialogue texts include:

- **Anonymization:** Efforts were made to anonymize the dialogues by removing names of individuals and locations, along with any personally identifiable or sensitive information, including remarks related to political or religious beliefs.
- **Elimination of non-essential details:** Segments of the patient exchanges that do not contribute to the main discussion, and instead inflate the character count (potentially complicating the summarization capabilities of certain models) are removed to prevent confusion and maintain focus on the primary subject matter. Fragments of non-essential details can be those that include information of a personal nature.
- **Reduction of word repetition:** The elimination of unnecessary repetition of words (whether from transcription inaccuracies or habitual repetition in spoken communication) aims to clarify communication, facilitating clearer distinction between speakers. An instance of this could be a patient affirming themselves with multiple “okay” utterances consecutively.

Following the initial processing phase, 14 out of the 30 documented discussions were chosen for their suitability for summarization, based on several critical factors. The selection criteria emphasized the legibility and coherence of the dialogues to ensure they could be easily understood. Preference was given to dialogues involving just two participants to minimize confusion about the source of statements. Additionally, the selection process favored discussions that were straightforward, with fewer complex topics and arguments, as these were easier to summarize accurately. Finally, dialogues that were overly lengthy, often correlating with higher complexity, were excluded to facilitate efficient summary information extraction.

### 3.5. Text Summarization

After processing the transcriptions, the next step involves summarizing these texts. This task will utilize models from Hugging Face capable of performing abstractive summarizations in Spanish. To avoid limiting the experiment to models that only accept Spanish inputs, which would exclude potentially more powerful models designed for English inputs, some English summarization models were also evaluated. Utilizing these English models necessitates an additional step of translating the Spanish transcriptions into English. Once translated, the transcriptions are fed into the summarization model being tested, and the resulting English summary is then translated back into Spanish using the *Helsinki-NLP/opus-mt-en-es* model, (https://huggingface.co/Helsinki-NLP/opus-mt-en-es, (accessed on 22 March 2024) also accessible through the Hugging Face API.

Many of these models have a maximum input text length capacity between 512 and 1962 characters, necessitating the division of pre-processed texts, which range from 4000 to 8000 characters, into smaller segments for analysis. In this study, two main approaches are considered in order to segment the text, distinguished by their methods of division and analysis:

- **Segmentation of processed transcriptions:** This method involves splitting the conversation at “\n” line breaks, with each segment then being processed on its own. During each cycle, the segment is tokenized with the tokenizer, followed by the generation of a summary through the use of a pre-trained model.
- **Segmentation in real time:** This approach employs a recursive method to break down the text into smaller pieces in case the length surpass the model’s capacity. Should the text prove too lengthy, it is divided into halves, with the procedure recursively applied to each segment. This repetition continues until the text size is manageable for the model’s processing capabilities.

Both strategies were applied to all the models under study, and the one which demonstrated a higher performance was selected for each of them. After experimenting with the different available models, a total of six were finally considered in the study. The rest were discarded because of poor results. These models are presented in Section 4.1.

## 4. Experiments

In this section, the experimentation developed is presented. First, the system is evaluated from a quantitative point and view and then from a qualitative one. In the quantitative approach, recorded conversations from the dataset explained in Section 3.2 are provided as input to the system proposed. The output produced by the system is then evaluated using two different metrics, which are explained in the next subsection. The experimentation evaluates the outputs of the system when different models are used for the text summarization component of the system. On the other hand, in the qualitative approach, an example of predicted summaries and their target is shown. Within this approach, the differences between predicted summaries and the variations with respect to the target summary are explained.

### 4.1. Quantitative Results

To obtain quantitative results, the Recall Oriented Understudy for Gisting Evaluation (ROUGE) metrics will be considered, which are the most widely used metrics to validate automatically generated summaries. ROUGE results will be presented in terms of two metrics: ROUGE-1, which evaluates the overlap of unigrams between the generated summary and the reference text, and ROUGE-L, which evaluates the overlap of the longest common word sequence between the generated summary and the reference text. Whereas ROUGE-1 provides a basic measure of lexical coherence and content relevance in the generated summary, ROUGE-L is a more complex metric, capturing content coherence and language fluency based on the longest word sequences. This considers the structure and order of words rather than being limited to unigrams.

In order not to limit the evaluation to lexicon similarity comparisons, the semantic-based metric BERTSCORE [34] is also included in one of the experiments in this study. BERTSCORE computes a semantic comparison of the summaries by comparing the contextual embeddings of the tokens of the summaries. Two experiments are conducted in this study. In the first, a wide variety of text summarization models are compared using ROUGE-1 and ROUGE-L metrics. Subsequently, the model exhibiting superior performance is then further evaluated using BERTSCORE and, finally, compared with two different high performance GPT models, which will be described in the introduction to the second experiment. As mentioned at the end of Section 3.5, for each of the models considered for the experimentations, ROUGE and BERTSCORE values are obtained using both text segmentation methods explained in that section, and the highest value is retained.

In this study, the six models that demonstrated superior performance on ROUGE metrics are shown. These models are as follows: *bart-large-cnn* (https://huggingface.co/facebook/bart-large-cnn, accessed on 22 March 2024) (BART), *distilbart-cnn-12-6* (https://huggingface.co/sshleifer/distilbart-cnn-12-6, accessed on 22 March 2024) (DISTILBART), *bart-large-cnn-samsum* (https://huggingface.co/philschmid/bart-large-cnn-samsum, accessed on 22 March 2024) (BART-SAMSUM), *gpt2* (https://huggingface.co/openai-community/gpt2, accessed on 22 March 2024) (GPT2), *bert2bert_shared-spanish-finetuned-summarization* (https://huggingface.co/mrm8488/bert2bert_shared-spanish-finetuned-summarization, accessed on 22 March 2024) (BERT2BERT), and *mT5_m2m_crossSum_enhanced* (https://huggingface.co/csebuetnlp/mT5_m2m_crossSum_enhanced, accessed on 22 March 2024) (T5). All the weights of these models have been extracted from the Hugging Face Transformers library, which is explained in more detail in the Section 3.1. Table 3 shows the average ROUGE metrics obtained from each of the models for the considered conversations in the dataset described in Section 3.2. As each conversation was complemented with two ground-truth summaries, the ROUGE metrics for each conversation were obtained by computing the average ROUGE between the two ground-truth summaries. See Table 4. Additionally, Table 5 presents the ROUGE scores for the conversation with the highest scores for each of the models.

As observed in the Table 3, ROUGE results are not as high as is desirable. The worst performance is observed when using the DISTILBART model, for which average results are 0.20 and 0.19 for ROUGE-1 and ROUGE-L, respectively. In contrast, the maximum average scores are achieved when using the BART-SAMSUM model, for which values ascend to 0.33 and 0.32. It is also remarkable that the GPT2 model obtains the absolute maximum values of 0.57 and 0.55, respectively, for one conversation. These limited results are partly due to the nature of the ROUGE metric, which is very sensitive to lexicon discrepancies between the ground truth and the generated summary. Even though the compared texts may be similar within the semantic scope, vocabulary variations between the reference text and the summary, such as the use of synonyms or paraphrased terms not present in the automatic summary, can significantly impact the effectiveness of ROUGE, thereby reducing the results obtained even though the meanings are similar. To demonstrate this fact, a second experimentation using not only the ROUGE metric but also a semantic-based metric is conducted.

In this second experiment, the model that showed the highest performance in the previous experiment (BART-SAMSUM) is fine-tuned using the dataset presented in Section 3.2. It is evaluated not only on the ROUGE-1 and ROUGE-L metrics but also on the BERTSCORE metric. BERTSCORE is a semantic-based metric that computes pairwise cosine similarity comparisons between all predicted and reference tokens to evaluate the similarity in the meanings of both summaries. Furthermore, the fine-tuned model is compared with two of the most recognized large language models, GPT-3.5 [35] and GPT-4 [36], to assess the trained model’s capabilities relative to current powerful models. To evaluate the models, three samples from the dataset are used, while the other eleven samples serve as training data. In the experiment, the pre-trained BART-SAMSUM model performance is compared with its fine-tuned version. The fine-tuned version of BART-SAMSUM is called BART-SAMSUM-F. Table 4 shows the results of this comparison.

The results illustrate the summarization capabilities of the BART-SAMSUM model when fine-tuning is applied. The GPT-3.5 and GPT-4 models demonstrate similar results with respect to the maximums presented in Table 3, achieving scores close to 0.32 in ROUGE-1 and 0.18 in ROUGE-L. However, the BART-SAMSUM model shows a decrease in performance compared to previous experiments. This decline is attributed to the reduced test set used in this experiment, which may include conversations that pose greater interpretation challenges to the model. Despite this, when the model is fine-tuned with the proposed dataset (BART-SAMSUM-F), the ROUGE metrics significantly improve, reaching 0.422 in ROUGE-1 and 0.271 in ROUGE-L, representing the highest scores achieved in the entire experiment and surpassing those of the GPT models, thus demonstrating its learning capabilities with limited data. As for the BERTSCORE, the results are less varied, with the minimum score of 0.66 when using BART-SAMSUM and the maximum of 0.73 obtained by BART-SAMSUM-F. These results demonstrate the capabilities of BART-SAMSUM-F in comparison to such powerful models as those in the GPT family. Furthermore, the BERTSCORE results suggest that, despite the use of different lexicons in the predicted and reference summaries, the underlying semantics are similar. This implies that the significance of the predicted summary aligns with that of the reference, indicating a similarity between both summaries.

The results of the proposed fine-tuned model, apart from being comparable with models from the GPT family, also align with the results obtained in other work related to the purposes of this study. In [21], a system fine-tuned on a dataset composed of almost 1700 dialogues achieves a maximum ROUGE-1 score of 0.425. In this cited work, a variant of BART [37] is used to compose the text summarization component of the system. On the other hand, in [22], a variant of the Longformer Encoder Decoder [38] model achieves a maximum ROUGE-1 score of 0.389, also being fine-tuned on a dataset of 1342 annotated conversations. The system proposed in our study manages to achieve a ROUGE-1 value of 0.422 using much more restrictive training data, which underscores the efficiency of the methods used and the capability of the framework to achieve very promising results in future research.

Although the experiment reached an acceptable BERTSCORE maximum of 0.735, the stringent accuracy requirements in healthcare settings, where mistakes can have serious consequences, currently make its deployment in these environments unviable. Nevertheless, the model’s demonstrated learning capabilities encourage further work on expanding the dataset to enable effective fine-tuning, which could significantly enhance the results.

### 4.2. Qualitative Results

To provide a broader view of the results obtained, an automatic summary generated by the proposed system is shown below, alongside its reference summary. This illustrates that, despite the low ROUGE scores, the main concepts of the transcribed conversation are accurately extracted. To compare the results of different models, the outputs of the two models that demonstrated the best performance on the ROUGE metric will be shown. Note that the original summaries are written in Spanish, but to facilitate understanding, the texts are translated into English. The reference summary is as follows:

“The patient comes in for a medical leave review following a car accident. The improvement is very slight and he is undergoing rehabilitation through the insurance company. The doctor recommends that he should not exert himself but should stay active. He reports pain in a shoulder. The doctor orders an X-ray and renews the medication that works well for the pain”.

Provided this reference summary, the automatic summary generated by *GPT2* is as folows:

“Okay, let’s go there. Did we have to update the report today? Yes. How are you doing? In pain. Now it seems that it hurts much less, but I still have the same pains and the dizziness is still the same. How was it? Because it was an accident, right? A car accident. Yes, it was a difficult accident. And what has the insurance told you? Or haven’t they told you anything? We are undergoing rehabilitation with the insurance. What do you do? I’m a maintenance technician. And do you use strength with your arms? Yes. Is it the right shoulder? Right shoulder, yes. And about medication? I need you to send me nolotil. What works best for you? I’m taking, sometimes I take nolotil, other times enentium. When you have the X-ray, if you want to see, or if we meet at the end of the month with the X-ray”.

On the other hand, the summary generated by *bart-large-cnn-samsum* is as follows:

“It was a car accident. The driver had to update the report today. He feels better now, but still has the same pains and dizziness. The dizziness takes at least a couple of months to go away. When I come out of rehabilitation, I have problems with my back. It’s important to stay active in rehabilitation and cook if you have to cook, so try to stay active and active. If you don’t do things that give you extra effort, but try to stay active, so if you need to cook then you cook. If the muscle relaxant at night works well for you, you can take it occasionally. If not, you have to take it less. If you don’t, then you can stop taking it altogether”.

Upon comparing the results, it is evident that both summaries capture the main topics of the conversation, which can be succinctly described as the nature of the accident (a car accident), the patient’s pain, and the prescribed medication. Whereas GPT2 produces a more extractive-based summary, where sentences in the summary are more likely to be directly taken from the transcription, *bart-large-cnn-samsum* delivers an abstractive summary, pinpointing main points and condensing all the information. Due to the extractive nature of GPT2, it also captures some secondary but relevant points. These points are related with doctor’s suggestion such as staying active, the mention of the insurance company, the specific location of the pain (the shoulder), and the reference to the X-ray test. On the other hand, *bart-large-cnn-samsum* is capable of removing conversational narration and transforming this into narrative text, aligning with the objective output of this study. Further research is necessary to retain the points extracted by GPT2 while generating a narrative summary similar to that provided by *bart-large-cnn-samsum*.

## 5. Conclusions

The study introduced an automated summary generation system tailored for healthcare professionals, intending to enhance care quality while alleviating bureaucratic workload. In the proposed approach, the input is first fed into an ASR model, which generates a transcription. Subsequently, the transcription undergoes a processing stage, and, finally, a Hugging Face Transformer model generates an abstractive summary based on the processed transcription.

The limitations in terms of data scarcity, which were insufficient for proper fine-tuning of the system, account for the low results obtained during experimentation when lexicon similarity is evaluated. Despite these low results, the results of the semantic-based evaluation and the qualitative comparison suggest that further research in the field, together with improvements to the system, could yield promising outcomes.

There are several potential avenues of future research that could improve the results obtained. These research lines include work such as researching methods aimed at enriching the medical terminology used in the generated summaries or using models already pretrained with medical data. Another area of future research, which is a crucial point in the experiments, is the expansion of the patient–doctor conversation dataset. Building a large conversation dataset with a wide variety of patients and situations can greatly improve the performance and reliability of the system.

## Figures and Tables

**Table 1:** Dataset statistics in terms of turns, sentences, and words.

Dialogues				Summaries	
	**Turns**	**Sentences**	**Words**	**Sentences**	**Words**
Total count	1702	1406	14,377	195	2061
Mean	122	100	1027	7	74
Maximum	225	144	1468	15	125

**Table 2:** Available Whisper models and their requirements. Data retrieved from https://github.com/openai/whisper (accessed on 22 March 2024).

Name	Parameters	VRAM Requirements	Speed
tiny	39 M	∼1 GB	∼32x
base	74 M	∼1 GB	∼16x
small	244 M	∼2 GB	∼6x
medium	769 M	∼5 GB	∼2x
large	1550 M	∼10 GB	∼1x

**Table 3:** Average ROUGE results for each of the models over the 14 conversations.

	ROUGE-1	ROUGE-L
BART	0.249	0.227
DISTILBART	0.201	0.186
BART-SAMSUM	**0.332**	**0.320**
GPT2	**0.332**	0.317
BERT2BERT	0.248	0.226
T5	0.272	0.248

**Table 4:** Comparison of *bart-large-cnn-sansum* model fine-tuned with respect to GPT3.5 and GPT4 models.

	ROUGE-1	ROUGE-L	BERTSCORE
BART-SAMSUM	0.099	0.072	0.666
GPT-3.5	0.325	0.186	0.719
GPT-4	0.319	0.182	0.697
BART-SAMSUM-F	**0.422**	**0.271**	**0.735**

**Table 5:** Maximum ROUGE results for each of the models in one of the conversations.

	ROUGE-1	ROUGE-L
BART	0.395	0.372
DISTILBART	0.372	0.319
BART-SAMSUM	0.558	0.485
GPT2	**0.574**	**0.553**
BERT2BERT	0.455	0.424
T5	0.424	0.383

## Data Availability

The dataset used during this study can be obtained by contacting the corresponding authors and agreeing to the license terms.
